# Evaluation of the Strategies to Control COVID-19 Pandemic in Four European Countries

**DOI:** 10.3389/fpubh.2021.700811

**Published:** 2021-10-05

**Authors:** Maria Michela Gianino, Mario Cesare Nurchis, Gianfranco Politano, Stefano Rousset, Gianfranco Damiani

**Affiliations:** ^1^Department of Public Health and Paediatrics, Università di Torino, Torino, Italy; ^2^Department of Woman and Child Health and Public Health, Fondazione Policlinico Universitario A. Gemelli IRCCS, Roma, Italy; ^3^Department of Control and Computer Engineering, Politecnico di Torino, Torino, Italy; ^4^Department of Health Sciences and Public Health, Section of Hygiene, Universit0 Cattolica del Sacro Cuore, Roma, Italy

**Keywords:** stringency index, indicators, containment measures, public health measure, COVID-19

## Abstract

On March 11, 2020, the World Health Organization (WHO) has officially declared the novel coronavirus outbreak a pandemic. The national governments deployed a series of severe control measures and a set of public health policies in order to stop the spread of COVID-19 pandemic. The aim of this study is to investigate the correlation between specific interventions and incident cases during the second wave in multiple and specific countries. The observational study was based on data from the Oxford COVID-19 Government Response Tracker (OxCGRT) source retrieved from October 1st, 2020 to January 10, 2021. Thirteen specific indicators related to measures adopted were considered. Four European countries were taken into account: Italy, German, Spain and UK. A vector autoregression (VAR) model and the Granger Causality test were performed to allow for an assessment of any possible effect induced by each control measure against the overall pandemic growth. Wald test was conducted to compute *p*-values. No correlation between the applied measures and incident cases in the four countries was shown by the Granger causality test. Only closings of workplaces (C2) and limits on private gatherings showed (C4) a significant correlation with incident cases in UK and restrictions on internal movement between cities/regions in Germany. The Granger causality also tested that C2 and C4 forecasted the decrease of incident cases after a time lag of 6–30 days in UK and Germany, respectively. Policy makers must analyze the context in which policies are set because of effectiveness of interventions can be influenced by local context and, especially, by socio-economic and demographic characteristics, and make a proper communication to support the resilience of the population capable of guaranteeing adherence to the interventions implemented.

## Introduction

On March 11, 2020, the World Health Organization (WHO) has officially declared the novel coronavirus outbreak a pandemic (1).

In most countries that have declared health emergencies to counteract the spread of coronavirus disease 2019 (COVID-19), measures have been adopted for the containment and management of the epidemiological emergency from COVID-19. Among the containment measures, defined as an intervention applied to a community in order to lower intermixing of unreported infectious individuals with susceptibles as well as the spread of the virus (2, 3), there are public health measures and government measures.

The national governments deployed a series of severe control measures (e.g., orders to stay at home, restricting travel, closing non-essential businesses, closing schools and other gathering places) and a set of public health policies (e.g., prevention and protection measures as washing or disinfecting hands with the appropriate disinfectant gel, using gloves, wearing a mask) aimed to curb the transmission of COVID-19 pandemic (4, 5).

The adoption of these measures occurs simultaneously in some countries while in others at different times, their maintenance is often discontinuous, and the application is carried out with different intensity. In addition, the measures that were adopted and how quickly they were embraced varied substantially—both across countries, and often within countries (4, 5).

These heterogeneities together with the specific political and socio-demographical contexts make the comparisons of the effectiveness of international pandemic responses complex.

However, the Oxford COVID-19 Government Response Tracker (OxCGRT) is a tool that allows for international comparison. This project provides a total of 19 indicators, of which eight are related to closures and containment measures, four to economic measures and seven to health measures. For each of them a different ordinal value indicates the score and, data is collected and updated in real time and reported daily (6).

Previous studies have investigated the effect of interventions on COVID-19 infection rates. Nevertheless, these studies either used composite measures – which combine different indicators into a general index (4) –, or inferred effectiveness of specific interventions, including social distancing (7) and travel restriction (8, 9), from a pool of countries or focused on a single country (10, 11). These studies are typically related to the first wave of the pandemic.

The aim of this study is to investigate the correlation between specific interventions and incident cases during the second wave in multiple and specific countries.

## Materials and Methods

Thirteen specific indicators were analyzed: eight were related to closures and containment measures while five to health measures. All of them are related to measures imposed to limit the transmission of COVID-19. Data of the 13 indicators, from October 1st, 2020 to January 10, 2021, were downloaded from the OxCGRT database (12) on January 15, 2021. Daily incident data on confirmed cases were used and retrieved from the OxCGRT database.

The indicators considered in the present study are shown in Table 1, which illustrates the definition, source and unit of measure for each item. Four European countries were taken into account: Italy, German, Spain and UK.

**Table 1:** Indicators, definitions, and coding^*^.

**Indicators**	**Definition**	**Coding**
C1	Record closings of schools and universities	0. no measures 1. recommend closing or all schools open with alterations resulting in significant differences compared to non-Covid-19 operations 2. require closing (only some levels or categories, e.g., just high school, or just public schools) 3. require closing all levels Blank - no data
C2	Record closings of workplaces	0. no measures 1. recommend closing (or recommend work from home) 2. require closing (or work from home) for some sectors or categories of workers 3. require closing (or work from home) for all-but-essential workplaces (e.g., grocery stores, doctors) Blank - no data
C3	Record canceling public events	0. no measures 1. recommend canceling 2. require canceling Blank - no data
C4	Record limits on private gatherings	0. no restrictions 1. restrictions on very large gatherings (the limit is above 1,000 people) 2. restrictions on gatherings between 101–1,000 people 3. restrictions on gatherings between 11–100 people 4. restrictions on gatherings of 10 people or less Blank - no data
C5	Record closing of public transport	0. no measures 1. recommend closing (or significantly reduce volume/route/means of transport available) 2. require closing (or prohibit most citizens from using it) Blank - no data
C6	Record orders to “shelter-in-place” and otherwise confine to the home	0. no measures 1. recommend not leaving house 2. require not leaving house with exceptions for daily exercise, grocery shopping, and 'essential' trips 3. require not leaving house with minimal exceptions (e.g., allowed to leave once a week, or only one person can leave at a time, etc.) Blank - no data
C7	Record restrictions on internal movement between cities/regions	0. no measures 1. recommend not to travel between regions/cities 2. internal movement restrictions in place Blank - no data
C8	Record restrictions on international travel Note: this records policy for foreign travelers, not citizens	0. no restrictions 1. screening arrivals 2. quarantine arrivals from some or all regions 3. ban arrivals from some regions 4. ban on all regions or total border closure Blank - no data
H1	Record presence of public info campaigns	0. no Covid-19 public information campaign 1. public officials urging caution about Covid-19 2. coordinated public information campaign (e.g., across traditional and social media) Blank - no data
H2	Record government policy on who has access to testing Note: this records policies about testing for current infection (PCR tests) not testing for immunity (antibody test)	0. no testing policy 1. only those who both (a) have symptoms AND (b) meet specific criteria (e.g., key workers, admitted to hospital, came into contact with a known case, returned from overseas) 2. testing of anyone showing Covid-19 symptoms 3. open public testing (e.g., “drive through” testing available to asymptomatic people) Blank - no data
H3	Record government policy on contact tracing after a positive diagnosis Note: we are looking for policies that would identify all people potentially exposed to Covid-19; voluntary Bluetooth apps are unlikely to achieve this	0. no contact tracing 1. limited contact tracing; not done for all cases 2. comprehensive contact tracing; done for all identified cases
H6	Record policies on the use of facial coverings outside the home	0. No policy 1. Recommended 2. Required in some specified shared/public spaces outside the home with other people present, or some situations when social distancing not possible 3. Required in all shared/public spaces outside the home with other people present or all situations when social distancing not possible 4. Required outside the home at all times regardless of location or presence of other people
H7	Record policies for vaccine delivery for different groups	0. No availability 1. Availability for ONE of following: key workers/clinically vulnerable groups/elderly groups 2. Availability for TWO of following: key workers/clinically vulnerable groups/elderly groups 3. Availability for ALL of following: key workers/clinically vulnerable groups/elderly groups 4. Availability for all three plus partial additional availability (select broad groups/ages) 5. Universal availability

* *Indicators, definitions and coding are reported as provided in Oxford COVID-19 Government Response Tracker (OxCGRT) raw data (12)*.

The original 13 indicators were loaded as time series in an R (13) custom pipeline and analyzed in terms of variance. Given the presence of a seasonal (weekly) pattern in confirmed daily cases, we performed a trend extraction by removing this high frequency seasonal noise to enforce the estimation reliability.

To assess any possible effect induced by each control measure against the overall pandemic growth, we resorted to the R *vars* package (14), which aims at estimation, forecasting and causality analysis by resorting to Vector autoregression (VAR) modeling to capture the relationship between multiple quantities as they change over time. To enforce and quantify the robustness of the causality estimation, we modeled the inference problem taking advantage of the Granger Causality test, thus fitting a VAR model and testing the hypothesis that the increase of any predictor Granger causes a decrease in infection growth. The *p*-value so far computed comes from a Wald test.

More in details: data has been transformed in a time-series (using function *ts* from {*stats*} package) with a weekly frequency (according to major weekly patterns present in data, i.e., recurrent reduction in numbers during week-end) then we applied a seasonal decomposition (*stl* function in {*stats*} package) to extract seasonal, trend and irregular components using a *periodic loess* interpolation.

Time lags have been automatically identified resorting to the *VARselect* function in {*vars*} package, which automatically increases the lag order up to 30 days. This limit has been chosen as a 25% percentage of the maximum time span of 4, 5 months available in raw data. Resorting to the best fitting time lag identified by VARselect we then computed the estimated VAR by utilizing OLS per equation (*VAR* function in {*vars*} package) and eventually computing the test statistics for Granger and Instantaneous causality for that VAR (*causality* function in {*vars*} package).

## Results

All the indicators C1, C2, C4, C7, C8, H3, were analyzed. The other indicators (i.e., C3, C5, C6, H1, H2) were not analyzed since the four considered countries deployed these interventions over the entire time span without any different intensity.

**Figure 1:**
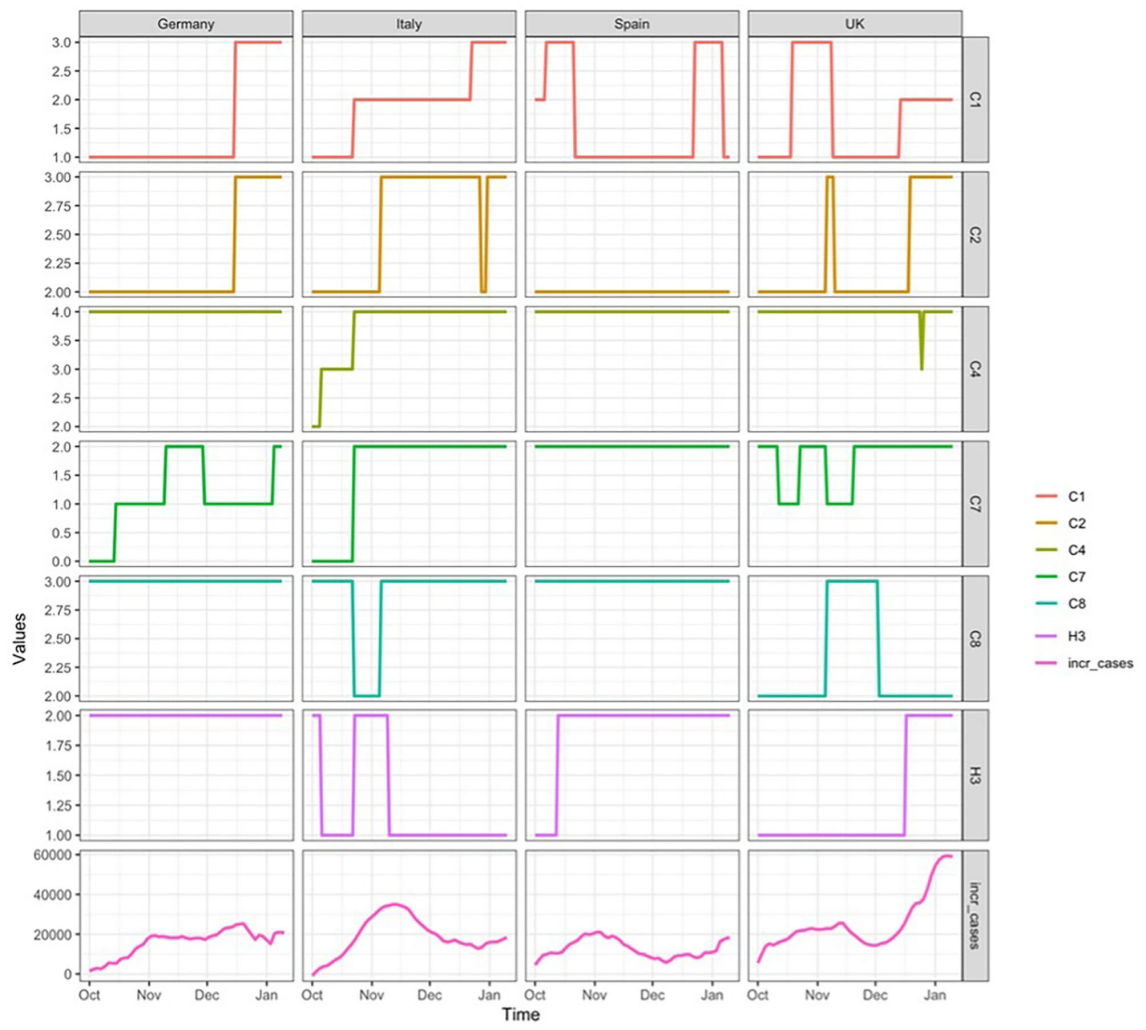
Values' trend of indicators and trend of incremental confirmed cases across the four European countries.

Figure 1 shows the values' trend of indicators and the trend of incremental confirmed cases across the four countries.

The Granger causality test (Table 2) showed that there was no correlation between the applied interventions and incident cases. Only C2 and C4 showed a significant correlation with incident cases in UK and C7 in Germany. The Granger causality also tested that C2 and C4 forecasted the decrease of incident cases after a time lag of 6 days in UK and C7 after 30 days in Germany. Other analyzed indicators did not show any significant correlation, thus they have been not reported in significant results.

**Table 2:** Results of the granger causality test and AIC days for four European countries (from 01/10/2020 to 10/01/2021).

**Ind**.	**Definition**	**Germany**		**Italy**		**Spain**		**UK**	
		***P*-value**	**AIC days**	***P*-value**	**AIC days**	***P*-value**	**AIC days**	***P*-value**		**AIC days**
C1	Record closings of schools and universities	N/A	29	0, 257	29	0, 648	30	0, 294		30
C2	Record closings of workplaces	N/A	29	0, 969	30	N/A	0	0, 042*		6
C4	Record limits on private gatherings	N/A	0	N/A	30	N/A	0	0, 014*		6
C7	Record restrictions on internal movement between cities/regions	0, 003*	30	N/A	30	N/A	0	0, 219		30
C8	Record restrictions on international travel	N/A	0	0, 719	30	N/A	0	0, 779		30
H3	Record government policy on contact tracing after a positive diagnosis	N/A	0	0, 984	30	N/A	28	0, 063		8

Ind. stands for Indicators. AIC stands for Akaike information criterion. P value* is significant at the 5% level. Where N/A is present the VAR algorithm was applied and showed no convergence, thus no results are available.

## Discussion

This study represents an interesting observational effort to evaluate the causality relationship of specific measures on incident cases. This approach is differentiated from previous studies that evaluated full down strategies (5, 15) or composite measures – which combine different indicators into a general index (4). Both of these approaches inevitably are abstract away from the significant nuance and heterogeneity exhibited by health policies' and Governments' responses. In addition, this study is an alternative approach to predictive studies that explore scenarios making strong assumptions that may be difficult to validate (9).

Our results pointed out that the most of specific interventions analyzed for Italy, German, Spain and UK are not correlated with incident cases of COVID-19. These results are in contrast to a previous study which showed that some interventions are effective to reducing transmission at the advent of the pandemic, highlighting that closing both schools and universities and limiting gatherings to 10 people produced a large effect while closing non-essential businesses produced a moderate effect (9). Anyhow, this study did not evaluate the effects across the countries.

Moreover, our results revealed that C2 e C4 have a Granger causality only in UK and C7 only in Germany.

The joint reading of our results leads to some suggestions.

First, like any policy intervention, the effect is likely to be highly contingent on local political and social contexts. A previous study (16) found that the effectiveness of many interventions depends on the local context and, in general, the effectiveness of social distancing measures and travel restrictions varies considerably across countries.

Second, the compliance with the measures may affect the spread of COVID-19. This suggestion is supported by evidence in the literature that identified demographic and socio-economic factors as variables able to influence the adherence with COVID-19 guidelines. According to previous studies, adolescents and young adults belong to a group that is the least likely to follow measures aimed at curbing the spread of COVID-2019, especially the recommended practices about hand-washing, self-isolation and social distancing (17–20).

A study in UK found that low compliance was strongly related to younger age, risk attitudes and high income, and that individuals living in overcrowded accommodation and neighborhoods with little space had lower and faster decreasing compliance (21). Another study in UK, justifying the Granger causality for C4 especially, tested that there was high compliance to social distancing and isolation guidelines reported across the study sample, and reported that lack of social conscience and lack of understanding a as likely causes of instances of non-adherence (22).

Additionally, the success of public health measures rests on the public's willingness to comply and the compliance with directives and recommended health behaviors is a longstanding and known problem (23). People routinely refuse to cease behavior that is bad for them and do not do what is good for them. This same pattern of behavior should be expected when it comes to COVID-19 restrictions being implemented. Just as people continue to smoke, to drink, to refuse to physical activity, to underestimate dietary risks, and even to reject required medication, so people will test the boundaries of government instructions, and many will simply refuse to comply.

Third, how and how well policies are enforced can affect the effectiveness of public health measures and government measures. To contain the COVID-19 pandemic, a critical decision is the extent to which policy makers rely either on voluntary or on enforced compliance. A survey conducted in Germany found that the effectiveness of voluntary compared with enforced measures to address the COVID-19 pandemic will differ across populations and suggests that enforcement might create less resistance in countries where trust in government is higher than in countries where trust in government is lower (10). In this way, an Italian study pointed out that, in Italy, the level of social and institutional trust is significantly low and Italians are known to live among relatives in large communities where close contact and deep personal interactions are the social glue. Consequently, due to the special familiar and relational structure and functioning of Italian society, the measures may be supported more under voluntary than under enforced implementation and the policy makers should have included risk communication measures able to educate and to encourage people to “*don't meet anyone* rather than merely *stay at home*” (24).

The results of this study and the subsequent preliminary conclusions must be considered in light of the study's weaknesses. The main limitations are those of the database used and are common to all administrative database studies. Firstly, there are problems related to the quality of the data which does not detect all significant nuance and heterogeneity of Governments' responses to COVID-19, this lack of granularity in stratification may results in a loss of sensitivity in identifying differences on some indicators. Secondly, OxCGRT includes data at country-level and, thus, the state-wide distribution was not analyzed. An analysis carried out within the national context may highlight particular local demographic, cultural, and socio-economical contexts able to affect policy intervention. Even taking these weaknesses into account, this database allows for systematic comparisons across countries. Finally, a further limit may be linked to the few number of states analyzed. However, these were chosen because they represent a broad range of public actions taken in response to pandemic in Europe.

In conclusion, although some studies (9, 17) show that some interventions such as closing school and restricting gathering places are effective to contain cases of COVID 19, the study' results demonstrate that specific interventions do not correlate with incident cases of COVID-19 in the four considered European countries. This raises the question of why similar policy measures appeared effective in stemming the outbreak in other countries, as China (11), and have had such a different impact across some European countries. Supported by the evidence in the scientific literature, this study suggested some hypotheses to answer the question that have implications for policy makers.

In the absence of perfect enforcement capacity by states, effectiveness of interventions can be influenced by local context and, especially, by socio-economic and demographic characteristics. On the other hand, the impact of interventions can be affected by cultural attitudes and behavioral norms.

This conclusion implies that policy makers must analyze more carefully the context in which the policies are set and consider that, in those countries where a level of enforcement similar to China may not be feasible, people must voluntarily comply with restrictions to be effective (25, 26).

The second implication, consistent with the literature (27, 28), regards the importance of taking care of communication so that people have a correct perception either of the risk of the pandemic or the benefits of restrictions. This is all the more so when it comes to individual mobility decisions, which entail a delicate trade-off between the chance of contracting (or diffusing) a disease and the costs associated with significant alterations of daily activities and that depends on the amount, quality, and interpretation of information available. The literature (29–31) highlighted that satisfaction with information received from the local government during an emergency seems to be related to the higher ability of an individual, group, or organization to continue its existence in the face of disaster such as pandemic.

Identifying the determinants of the effectiveness of adopted measures was not the aim of this study, thus, further research is needed on factors that influence the effects of control measures and public health policies to corroborate our implications.

## Data Availability Statement

Publicly available datasets were analyzed in this study. This data can be found here: https://covidtracker.bsg.ox.ac.uk/about-api.

## Author Contributions

MG and GD: conceptualization, validation, and supervision. MN and SR: data curation. GP: formal analysis. MN, MG, and GD: methodology. MG: writing—original draft. MN, MG, and GD: writing—review and editing. All authors contributed to the article and approved the submitted version.

## Conflict of Interest

The authors declare that the research was conducted in the absence of any commercial or financial relationships that could be construed as a potential conflict of interest.

## Publisher's Note

All claims expressed in this article are solely those of the authors and do not necessarily represent those of their affiliated organizations, or those of the publisher, the editors and the reviewers. Any product that may be evaluated in this article, or claim that may be made by its manufacturer, is not guaranteed or endorsed by the publisher.
